# 4543 Glucocorticoid Receptors are essential effectors of TGFβ signaling in Triple Negative Breast Cancer

**DOI:** 10.1017/cts.2020.68

**Published:** 2020-07-29

**Authors:** Carlos Jesus Perez Kerkvliet, Amy R Dwyer, Caroline Diep, Robert Oakley, Christopher Liddle, Carol A Lange

**Affiliations:** 1 University of Minnesota CTSI

## Abstract

OBJECTIVES/GOALS: The glucocorticoid receptor (GR) is a ubiquitous steroid hormone receptor that is emerging as a mediator of breast cancer metastasis. We aim to better understand the biology associated with phospho-GR species in TNBC and their contribution to tumor progression. METHODS/STUDY POPULATION: To better understand how p-S134 GR may impact TNBC cell biology, we probed GR regulation by soluble factors that are rich within the tumor microenvironment (TME), such as TGFβ. TNBC cells harboring endogenous wild-type or S134A-GR species were created by CRISPR/Cas knock-in and subjected to *in vitro* assays of advanced cancer behavior. RNA-Seq was employed to identify pS134-GR target genes that are uniquely regulated by TGFβ in the absence of exogenously added GR ligands. Direct regulation of selected TGFβ-induced pS134-GR target genes was validated accordingly. Bioinformatics tools were used to probe publicly available TNBC patient data sets for expression of a pS134-GR 24-gene signature. RESULTS/ANTICIPATED RESULTS: In the absence of GR ligands, GR is transcriptionally activated via p38-MAPK-dependent phosphorylation of Ser134 upon exposure of TNBC cells to TME-derived agents (TGFβ, HGF). The ligand-independent pS134-GR transcriptome primarily encompasses gene sets associated with TNBC cell survival and migration/invasion. Accordingly, pS134-GR was essential for TGFβ-induced TNBC cell migration, anchorage-independent growth in soft-agar, and tumorsphere formation, an *in vitro* readout of breast cancer stemness properties. Finally, a 24-gene pSer134-GR-dependent signature induced by TGFβ1 predicts shortened survival in breast cancer. We expect to find similar results using an in-house tissue microarray. DISCUSSION/SIGNIFICANCE OF IMPACT: Phospho-S134-GR is a critical downstream mediator of p38 MAPK signaling and TNBC migration, survival, and stemness properties. Our studies define GR as a required effector of TGFβ1 signaling and nominate pS134-GR as a biomarker of elevated risk of breast cancer dissemination.

